# Three-dimensional phase-contrast X-ray microtomography with scanning–imaging X-ray microscope optics

**DOI:** 10.1107/S0909049513018876

**Published:** 2013-07-26

**Authors:** Akihisa Takeuchi, Kentaro Uesugi, Yoshio Suzuki

**Affiliations:** a Research and Utilization Division, Japan Synchrotron Radiation Research Institute, 1-1-1 Kouto, Sayo, Sayo-gun, Hyogo 679-5198, Japan

**Keywords:** X-ray microscopy, differential phase contrast, tomography

## Abstract

A three-dimensional (3D) X-ray tomographic micro-imaging system has been developed. The optical system is based on a scanning–imaging X-ray microscope (SIXM) optics, which is a hybrid system consisting of a scanning microscope optics with a one-dimensional (1D) focusing (line-focusing) device and an imaging microscope optics with a 1D objective. In the SIXM system, each 1D dataset of a two-dimensional (2D) image is recorded independently. An object is illuminated with a line-focused beam. Positional information of the region illuminated by the line-focused beam is recorded with the 1D imaging microscope optics as line-profile data. By scanning the object with the line focus, 2D image data are obtained. In the same manner as for a scanning microscope optics with a multi-pixel detector, imaging modes such as phase contrast and absorption contrast can be arbitrarily configured after the image data acquisition. By combining a tomographic scan method and the SIXM system, quantitative 3D imaging is performed. Results of a feasibility study of the SIXM for 3D imaging are shown.

## Abstract 2

A novel three-dimensional X-ray microtomographic micro-imaging system which enables simultaneous measurement of differential phase contrast and absorption contrast has been developed. The optical system consists of a scanning microscope with one-dimensional focusing device and an imaging microscope with one-dimensional objective.

## 1. Introduction   

Phase-contrast imaging in the hard X-ray region is well known as having a much higher sensitivity than absorption-contrast imaging especially for low-*Z* materials. Even for high-*Z* materials, phase-contrast micro-imaging is more suitable than absorption-contrast imaging as observable objects are becoming smaller with progress in the spatial resolution of the X-ray imaging technique. Therefore, various types of phase-contrast X-ray microscope have been developed in the last few decades. In particular, many approaches implemented in imaging (full-field) X-ray microscope optics for phase-contrast and differential phase-contrast imaging have been proposed such as Zernike phase contrast (Schmahl *et al.*, 1994 ▶; Kagoshima *et al.*, 2001 ▶), Talbot interferometers (Takeda *et al.*, 2008 ▶; Yashiro *et al.*, 2009 ▶) and two-beam interferometers (Suzuki & Takeuchi, 2005 ▶; Suzuki *et al.*, 2010 ▶; Watanabe *et al.*, 2006 ▶; Koyama *et al.*, 2004 ▶, 2006 ▶). On the other hand, the generation of differential phase contrast in scanning X-ray microscopy has also been widely studied (Chapman *et al.*, 1995 ▶; Morrison *et al.*, 2003 ▶; Takano *et al.*, 2003 ▶; Hornberger *et al.*, 2007 ▶; Kagoshima *et al.*, 2004 ▶). In such a system, position-sensitive X-ray detectors are used, while a simple X-ray counter is used in conventional scanning microscopes.

The advantages and disadvantages of these two optical systems are complementary to each other. One of the advantages of the imaging microscope optics is high throughput. One of the disadvantages is that it is difficult to realise both high-quantitative and high-sensitive imaging. For example, Zernike phase-contrast imaging is not suitable for quantitative phase measurement although it is highly phase sensitive. Differential phase-contrast imaging with a Talbot interferometer enables a quantitative phase measurement; however, sensitivity is not sufficient for highly magnified imaging. The two-beam interferometer method which realises both a high quantitativity and a high sensitivity requires stringent stability conditions; however, it is difficult to sustain stability for a long time measurement such as in tomography. Another disadvantage is the relatively high radiation dose because of the loss of transmitted X-ray intensity, especially for an objective with low efficiency such as a thin Fresnel zone plate. On the other hand, advantages of scanning optics are quantitative phase measurement, high phase sensitivity and low dose. The most serious disadvantage is low throughput. Therefore, three-dimensional (3D) imaging applications have not yet been in practical use because full 3D data acquisition requires a lot of time even with state-of-the-art systems which enable millisecond dwell time scans (Hornberger *et al.*, 2008 ▶; Takeuchi *et al.*, 2010 ▶).

**Figure 1:**
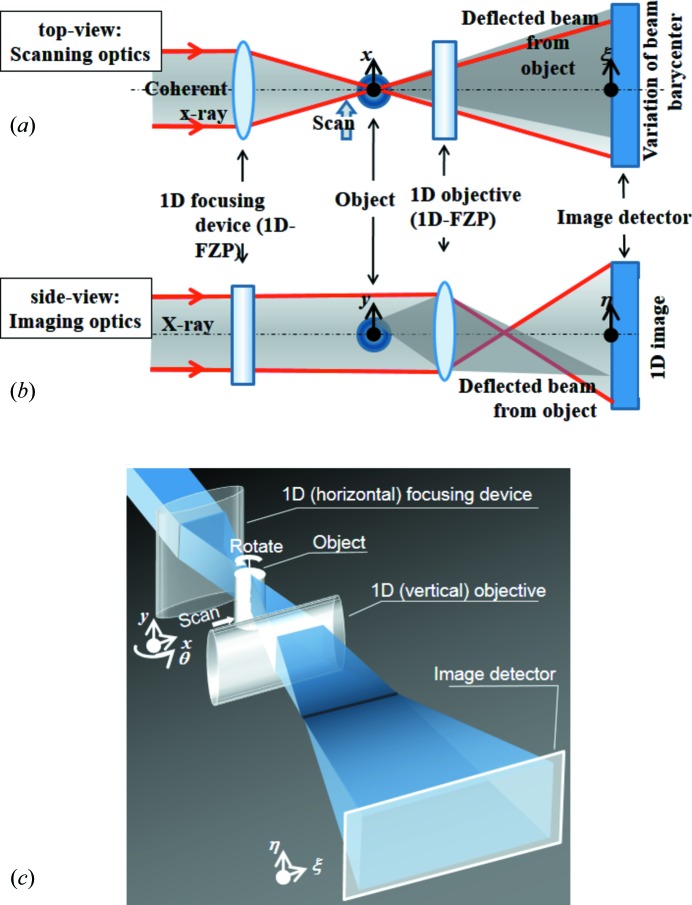
Conceptual drawing of the scanning–imaging X-ray microscope (SIXM). (*a*) Top-view, (*b*) side view, (*c*) bird’s-eye view.

In order to employ the data acquisition technique of differential phase-contrast scanning microscopy for practical 3D imaging applications, we have developed an optical system combining a differential phase-contrast scanning microscope optics and an imaging microscope optics (scanning–imaging X-ray microscope, SIXM). This concept satisfies the advantages of these two systems such as high sensitivity and high quantitativity of differential phase-contrast scanning optics and high throughput of imaging optics. A conceptual drawing of the optical system of the SIXM is shown in Fig. 1 ▶. The top view and side view of the system are completely different from each other. In the top view the system is the same as the scanning microscope optics. A one-dimensional (1D) focusing device is used to produce a line-focused probe (in this case, horizontal focus) onto the object plane. In the side view, on the other hand, the system is the same as the imaging microscope. A 1D objective is installed between the object plane and the image plane, keeping the conditions of the lens formula to obtain enlarged vertical positional information of the object. In the SIXM system the line-focus optics and the image-forming optics are set normal to each other with regard to their common optical axis. Positional information of the object region illuminated with the line-focused beam is recorded simultaneously with the 1D imaging microscope as line-profile data. By scanning the sample with the line-focused beam, a two-dimensional (2D) image dataset is obtained. A 2D imaging detector is placed in the image plane. The detector is used as an imaging detector for the imaging optics, and as a position-sensitive detector for the scanning optics that enables quantitative differential phase contrast (and absorption contrast) to be measured in the same manner as a conventional scanning microscope with multi-pixel detector.

Raw data obtained in the image plane (ξ, η) consists of sequential image data *I*(ξ, η; *x*_*a*_) where *x*_*a*_ represents the displaced distance of an object at the object plane (*x*, *y*) by scanning translation. Horizontal and vertical elements of *I* independently represent the far-field distribution of the horizontally focused probe beam and the vertically magnified inverted image of the object, respectively. Therefore, image data acquisition processes are independent of each other between the horizontal direction and the vertical direction.

In the scanning microscope optics, by translating the object in the horizontal direction a sequence of far-field image data of the transmitted beam is acquired with the imaging detector at each point in the scan. When the object is located at a position *x*_*a*_ on the object plane, the image signal of the scanning microscope optics *s*(*x*_*a*_) is given by
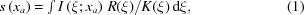
where *I*(ξ; *x*_*a*_) is the horizontal distribution of measured X-ray intensity at the detector plane, *R*(ξ) is the detector response function configured according to the intended image-contrast mode (Morrison *et al.*, 2003 ▶), and *K*(ξ) is a compensation factor introduced for the compensation of unevenness of the optical transfer response (Takeuchi *et al.*, 2012 ▶). If the sensitivity of the detector pixel is uniform and the optical transfer function of the focusing device is ideal, *K*(ξ) = 1 for every ξ.

On the other hand, in the vertical direction, a 1D objective device is installed between the object and the image detector, satisfying Newton’s lens equation. When a detector plane is at a conjugate of the objective plane, the vertical intensity distribution is expressed as
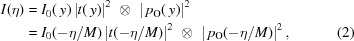
where 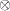 denotes the convolution operator, *I*_0_(*y*) is the intensity distribution at the object plane without sample, *t*(*y*) is the complex amplitude transmission function of the object, |*p*_O_(*y*)|^2^ is the line-spread function of the objective, and *M* is the magnification factor of the imaging microscope optics. If the line-spread function |*p*_O_(*y*)|^2^ can be assumed to be a delta function, (2) is given as
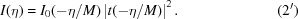
This equation denotes a similarity relationship between the object and the image with the magnification factor −*M*. Taking (2′) and (1) into account, the vertical line profile of the object at the horizontally displaced distance in the object plane *x*_*a*_ is given as
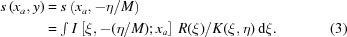
By scanning a sample in the horizontal (*x*) direction, 2D image data *s*(*x*, *y*) are acquired. By using a multi-pixel detector such as a CCD array, imaging modes such as absorption contrast and partial differential phase contrast can be arbitrarily derived from the acquired data. In an absorption-contrast scanning microscope geometry, the configuration of the response function is *R*(ξ) = 1. In this case, (3) can be rewritten as
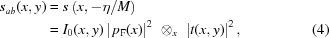
where 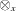 denotes a convolution with respect to *x*, and *p*_F_(*x*) is the probe function of the focusing device. On the other hand, in the case of the partial differential phase-contrast mode, the configuration of the response function is *R*(ξ) = ξ. For the complex transmission function *t*(*x*, *y*) given by *t*(*x*, *y*) = exp[α(*x*, *y*) + *i*ϕ(*x*, *y*)], the image signal is given as
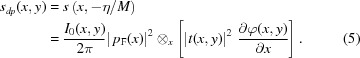
The partial phase gradient ∂ϕ/∂*x* is experimentally derived by measuring the deviation of the transmitting beam barycenter which is equivalent to *s*_*dp*_(*x*, *y*)/*s*_*ab*_(*x*, *y*).

Since a 2D image can be obtained in a single translation scan, while the conventional scanning optics requires a 2D raster scan, a significant reduction in measurement time is expected.

## 2. Experimental set-up   

The experiment was carried out at the undulator beamline 20XU of SPring-8. The X-ray source was an in-vacuum-type permanent-magnet undulator with a periodic length of 26 mm and a period of 173 installed in the 8 GeV electron storage ring of SPring-8. The undulator radiation was monochromated to 8 keV with monochromaticity Δ*E*/*E* ≃ 10^−4^ by passing through a liquid-nitrogen-cooled Si(111) double-crystal monochromator located at 45 m from the source. The monochromatic beam was transported through a 200 m-long vacuum beam duct, and the experimental station in which the experiment was performed was located at 245 m from the light source. The effective source size in the vertical direction was approximately 40 µm full width at half-maximum (FWHM), determined by the electron beam size of the storage ring and by the vibration of the monochromator crystal, estimated to be approximately 0.1 arcsec. Although the horizontal source size is exactly 700 µm FWHM, in this experiment the horizontal effective source size was defined by a water-cooled front-end slit located at ∼32 m from the source. The opening size of the front-end slit was set to 500 µm in the vertical (V) direction × 100 µm in the horizontal (H) direction. Therefore, the effective source size was assumed to be 40 µm (V) × 100 µm (H). Details of the beamline have already been described elsewhere (Suzuki *et al.*, 2004 ▶). Since the spatial coherence at the experimental hutch was approximately 1 mm (V) × 0.4 mm (H), much larger than the aperture size of the optical devices (0.155 mm), the system was in full-coherent illumination mode.

**Figure 2:**
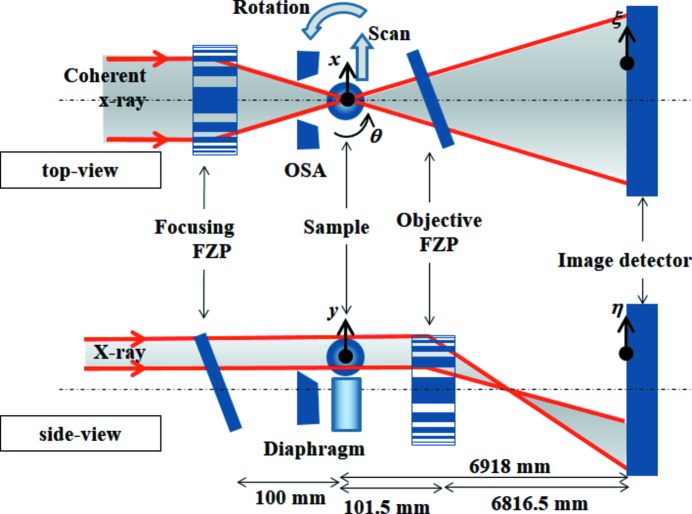
Schematic diagram of the experimental set-up of the SIXM-CT system at BL20XU experimental hutch 2 of SPring-8 (top: top view; bottom: side view). A high-accuracy rotation stage for tomographic scans is installed in the sample plane such that the rotation axis is along the *y*-axis. FZP: Fresnel zone plate; OSA: order-sorting aperture. The focal length of the focusing FZP *f* is 100 mm for 8 keV X-rays.

The experimental set-up of the SIXM-CT system is shown in Fig. 2 ▶. The set-up consisted of a scanning computed tomography (CT) system for the horizontal direction and imaging optics for the vertical direction.

A pair of 1D Fresnel zone plates (FZPs) with the same parameters was used as the 1D focusing device and as the 1D objective. The FZPs were fabricated at NTT-AT using the electron-beam lithography and reactive-ion etching technique (Sekimoto *et al.*, 1988 ▶; Ozawa *et al.*, 1997 ▶). A tantalum zone pattern is deposited on a silicon carbide membrane of thickness 2 µm. The outermost zone width of the FZPs is 100 nm, the width of the FZP pattern is 155 µm, and the tantalum zone thickness is 1 µm. The focal distance at an X-ray energy of 8 keV is 100 mm. The measured efficiency of the first-order diffraction at 8 keV X-ray energy was 0.168 while the theoretical value for the 1 µm-thick tantalum is 0.189. In the experiment, the FZPs were set inclined by 60° in the perpendicular to their respective zone directions in order to increase the effective zone thickness to twice the original value. Under this condition, the measured value of the diffraction efficiency was 0.279 (the theoretical value is 0.316), maintaining the condition of diffraction-limited spatial resolution (Takeuchi *et al.*, 2012 ▶). As with a typical imaging microscope optics, an increase in the diffraction efficiency of the objective results in a reduction of the radiation dose and an increase in intensity. Because each 1D FZP was set inclined, the focal plane was also inclined to the object plane. Therefore, the effective field of view (FOV) in the object plane is restricted by the focal depth. In the case of the present SIXM system in which both 1D FZPs were inclined, the effective FOV of each of the scanning and imaging optics was restricted by the other. The relation between the FOV of one and the depth of focus (Δ*f*) of the other can be expressed by FOV ≤ Δ*f*/tanθ, where θ is the inclination angle. Assuming that the system performs in a diffraction-limited manner, the depth of focus is given by Δ*f* = λ/(2NA^2^) where λ is the wavelength and NA is the numerical aperture. In the present case where the inclined angle θ = 60°, since the depth of focus is Δ*f* = 129 µm for λ = 0.155 nm and NA = 7.75 × 10^−4^, then the available FOV is calculated to be 74.5 µm.

In the imaging optics, a (coherent) quasi-parallel illuminating system was employed. In this case an objective FZP was required to be used under the off-axis condition for the diffraction order sorting of the FZP. A three-jaw tantalum slit, consisting of two blades to define the horizontal beam and a blade to cut the lower region of the beam, was installed just in front of the object plane. An opening size in the horizontal direction of 20 µm was used as an order-sorting aperture for the focusing FZP. In the vertical direction, this slit was used as a diaphragm of the objective FZP. In order to eliminate overlapping of the third-order diffraction of the objective at the image plane, the paraxial region of more than one-third of the half-aperture size of the objective FZP must be blind. In the present experiment, the slit edge was set overhanging the optical axis by 30 µm, *i.e.* 38.7% of the half-aperture width of the FZP (77.5 µm). Therefore, a paraxial region of ±30 µm was blind.

A stepping-motor-driven translation stage (Kohzu Precision) was used for the horizontal sample scan. A high-precision rotation stage with a wobbling of less than 100 nm (Kohzu Precision) was used as the sample rotation stage for the tomographic scan. The rotation stage was set so that the rotation axis was along the vertical direction. Therefore, three independent directions of a 3D-CT image dataset such as the rotational direction, radial direction and vertical direction are measured by the tomographic scan (θ), the scanning optics (*x*) and the imaging optics (*y*), respectively.

A helium-flow duct with polyimide film windows of thickness 25 µm was installed between the objective FZP and the image detector in order to reduce the X-ray attenuation by air. Two FZPs were also set in helium-flow ducts with silicon nitride membrane windows of thickness 2 µm to reduce the radiation damage. These chambers were always flowed with helium gas of purity higher than 99.8% during the experiment.

An indirect-sensing X-ray camera consisting of a visible-light conversion unit called the beam monitor (BM AA40P, Hamamatsu Photonics) and a frame-transfer-type CCD camera (C9100-02, Hamamatsu Photonics) were used as the image detector. The beam monitor consisted of a P43 powder scintillator (Gd_2_O_2_S:Tb, 10 µm thickness) for conversion from an X-ray image to a visible-light image, and a couple of lenses with focal lengths of 50 mm (Nikon) and 35 mm (Nikon). Typical parameters of the CCD camera are as follows: pixel size, 8 µm; number of pixels, 1000 × 1000; maximum frame rate for full frame, 30 Hz; analog–digital converter, 14-bit. Since the magnification of the lens system of the beam monitor is 0.7× (= 35/50), the effective pixel size of the image detector is 11.4 µm, and the detective area is 11.4 mm × 11.4 mm.

The distance between the object plane and the objective FZP was 101.5 mm and the distance between the object plane and the image plane was ∼6918 mm. Therefore, the magnification factor of the X-ray imaging microscope optics was approximately 67, and the converted pixel size at the object plane was approximately 172 nm. The width of the far-field image at the detector plane was approximately 10.9 mm.

**Figure 3:**
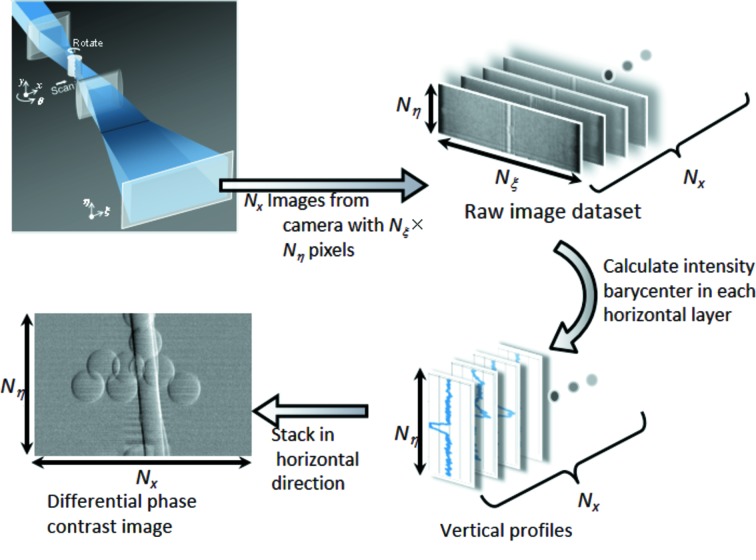
Workflow of differential phase-contrast 2-D image acquisition from a raw image dataset.

The workflow of the 2D image acquisition process from the acquisition of a raw image dataset is shown in Fig. 3 ▶. Raw images *I*(ξ, η; *x*_*a*_) with number of pixels *N_ξ_* × *N_η_* are recorded with the image detector for every point in the horizontal scan. In the raw image, horizontal and vertical elements represent the far-field distribution of the focused beam and the inversely enlarged image formed with the objective, respectively. The same number of raw images as the number of scan points (*N*_*x*_) is recorded in one translation scan. For each raw image data, a vertical line profile is derived using equation (3), employing a proper form of *R*(ξ) (the case of the reconstruction of differential phase-contrast mode is represented in Fig. 3 ▶). By stacking *N*_*x*_ vertical profiles in series in the horizontal direction, a 2D image with number of pixels *N*_*x*_ in the horizontal × *N*_η_ in the vertical is reconstructed. Horizontal and vertical pixel sizes of the reconstructed image are equivalent to the horizontal scan pitch of the object and the converted pixel size of the imaging microscope optics, respectively. For the tomographic scan, these processes are repeated for each rotation angle. Therefore, a raw image dataset consists of *N*_ξ_ × *N*_η_ × *N*_*x*_ × *N*_θ_ data points for one tomographic scan with *N*_θ_ rotation steps.

## 3. Results   

### 3.1. Polystyrene spheres   

**Figure 4:**
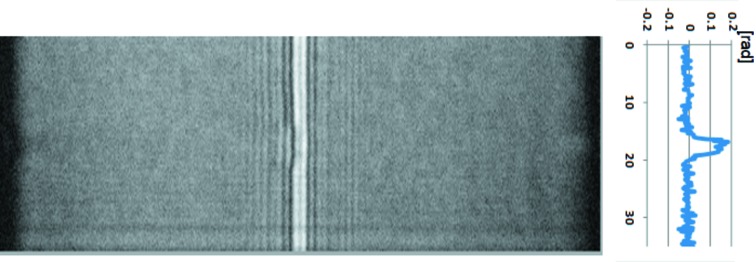
Raw image data of the SIXM-CT system obtained when the line-focused beam is located at the line *AB* shown in Fig. 5(*a*) ▶. Vertical line profiles of differential phase shift ∂*f*/∂*x* derived from the raw data by using equation (5) are also indicated on the right-hand side. Exposure time is 60 ms.

**Figure 5:**
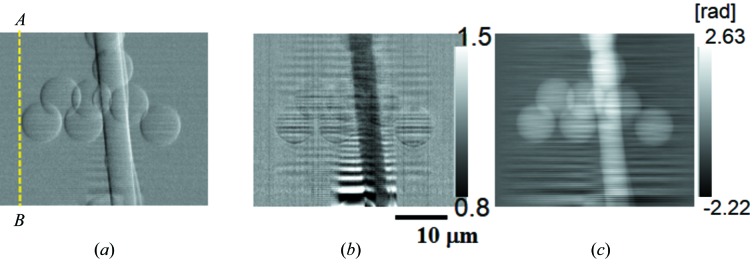
Two-dimensional X-ray images of polystyrene spheres (8 µm diameter) stuck on a glass fiber (5 µm diameter). (*a*) Differential phase-contrast image, (*b*) absorption-contrast image and (*c*) phase-contrast image obtained by the horizontal linear-integration of the differential phase-contrast image (*a*). Pixel size and the number of pixels of the reconstructed images are 175 nm (H) × 172 nm (V) and 309 (H) × 208 (V), respectively. Horizontal scan pitch is 175 nm, exposure time is 60 ms, dwell time is 66.7 ms and measurement time is approximately 20 s.

Feasibility tests for 3-D imaging were performed using a sample consisting of polystyrene spheres of diameter 8 µm stuck on a silica glass fiber of diameter 5 µm. Fig. 4 ▶ shows one of the raw image datasets *I*(ξ, η; *x*_*a*_). The vertical line profile of the differential phase shift ∂ϕ/∂*x* derived from the raw data by using equation (5) is also indicated on the right-hand side in Fig. 4 ▶. This raw image was obtained when the line-focused probe beam was located at the left-hand edge of the polystyrene sphere shown as the line *AB* in Fig. 5(*a*) ▶. In the raw image data of Fig. 4 ▶, signal modification in the horizontal direction is seen at the vertical position where the probe is in contact with the sphere. As a result, this phenomenon is recognized as a phase gradient ∂ϕ/∂*x* in the line profile.

Figs. 5(*a*) and 5(*b*) ▶ show the X-ray 2-D differential phase-contrast image and the absorption-contrast image reconstructed from the raw dataset (Fig. 4 ▶) by using equations (5) and (4), respectively. A flat-field correction is processed using the left-end region of the image where no sample exists. Fig. 5(*c*) ▶ shows a phase-contrast image reconstructed from the differential phase-contrast image (Fig. 5*a* ▶) by processing a linear integration along the horizontal direction. Upper and lower regions of these images correspond to the paraxial region and the periphery of the field of view, respectively. Since the object consists of low-*Z* materials, the object is much more clearly observed in the differential phase-contrast and phase-contrast images than with the absorption contrast at 8 keV.

In the phase-contrast image (Fig. 5*c* ▶), horizontally streaking white noise can be seen. This is typical in the linear integration reconstruction process of differential phase-contrast optics. In addition, another type of horizontally streaking background noise, called ringing, is seen in each image, particularly in the lower region of the images. This will be discussed in the next chapter.

**Figure 6:**
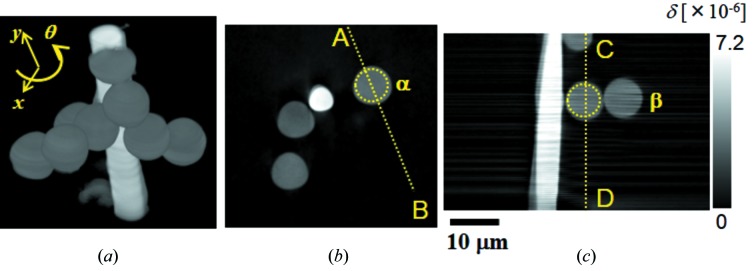
Phase-contrast CT images of polystyrene spheres (8 µm diameter) pasted on a glass fiber (5 µm diameter). (*a*) 3D rendered view, (*b*) virtual cross-sectional CT image in the horizontal plane perpendicular to the rotation axis and (*c*) virtual cross-sectional CT image in the vertical direction parallel to the rotation axis. Voxel size is 175 nm × 175 nm (H) × 172 nm (V). Horizontal translation scan pitch is 175 nm, exposure time is 30 ms and dwell time is 33.3 ms. Number of projections is 501 for 180° rotation. Total measurement time for a tomographic scan is approximately 200 min.

**Figure 7:**
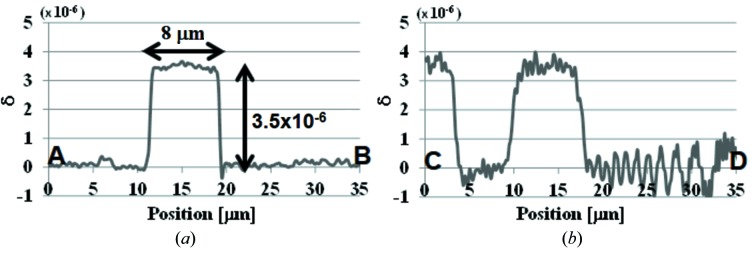
Line profiles in phase-contrast CT images corresponding to (*a*) the line *AB* in Fig. 6(*b*) and (*b*) the line *CD* in Fig. 6(*c*) ▶.

Phase-contrast CT images are shown in Fig. 6 ▶. Figs. 6(*a*), 6(*b*) and 6(*c*) ▶ correspond to a 3-D rendered view and virtual cross-sectional images in the horizontal plane (perpendicular to the rotation axis) and the vertical plane (parallel to the rotation axis), respectively. The voxel value represents the decrement of the refractive index from unity expressed by δ. Line profiles on the line *AB* in Fig. 6(*b*) ▶ and the line *CD* in Fig. 6(*c*) ▶ are shown in Figs. 7(*a*) and 7(*b*) ▶, respectively. The voxel value of the polystyrene is measured to be ∼3.5 × 10^−6^ as shown in Fig. 7 ▶. This is in a good agreement with the theoretical value of δ = 3.676 × 10^−6^. Owing to the horizontally streaking noise mentioned above, the signal-to-noise ratio of the CT image is different between the horizontal plane and the vertical plane. This is confirmed by comparing the line profiles in Figs. 7(*a*) and 7(*b*) ▶ which show the CT image contrast in the horizontal plane and in the vertical plane, respectively. Fig. 7(*b*) ▶ shows not only a poorer noise level than Fig. 7(*a*) ▶ but also the ringing noise whose amplitude is increasing towards the right-hand side [corresponding to the lower region of the image of Fig. 6(*c*) ▶]. The standard deviation of δ in the horizontal cross section is measured from the region surrounded by the circle α in Fig. 6(*b*) ▶ to be 9.0 × 10^−8^. Therefore, the density resolution in the horizontal direction estimated with threefold of the standard deviation is approximately 79 mg cm^−3^, where an approximate expression between δ and the density ρ as δ = 1.35 × 10^−6^ρλ^2^ [g cm^−3^ Å] (δ = 3.5 × 10^−6^ and ρ = 1.05 g cm^−3^ for polystyrene) is used. On the other hand, the standard deviation of δ and the density resolution in the vertical cross section measured from the region surrounded by the circle β in Fig. 6(*b*) ▶ are approximately 3.0 × 10^−7^ and 265 mg cm^−3^, respectively. Although the density resolution in the vertical direction may be different according to the measured region because of the different amplitude of the ringing noise, these results show that image contrast is much different between the horizontal direction and the vertical direction.

### 3.2. Fossil of a diatom   

**Figure 8:**
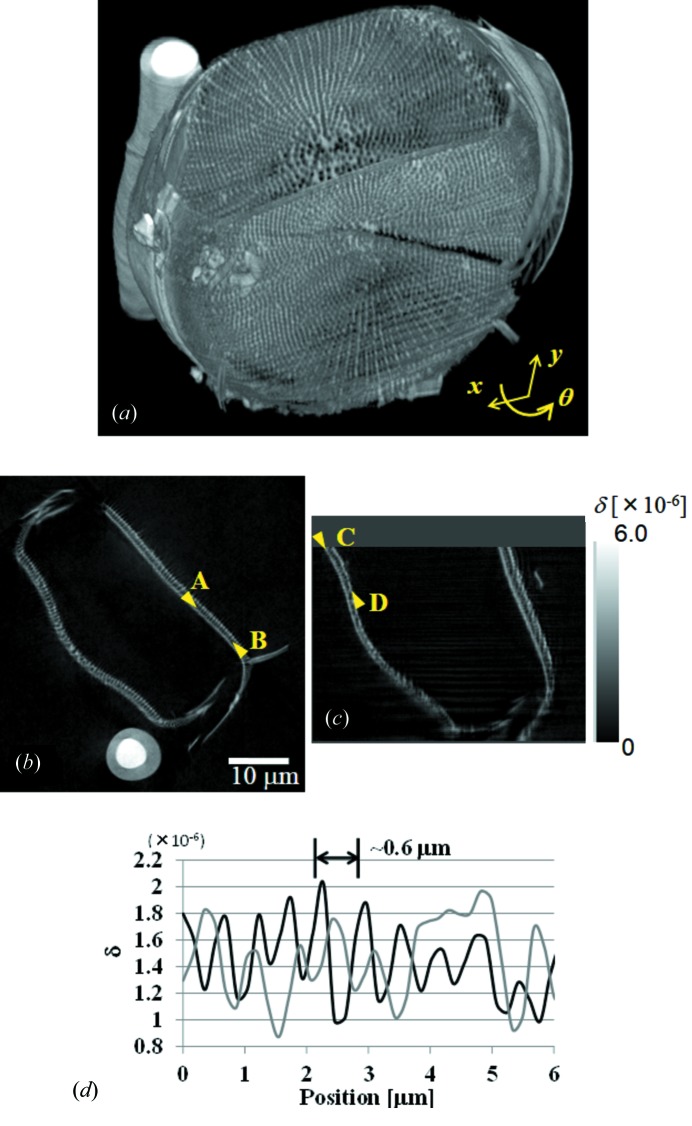
X-ray phase-contrast CT images of a diatom fossil pasted on a glass fiber. (*a*) 3D rendered view, (*b*) virtual cross-sectional image perpendicular to the rotation axis, (*c*) virtual cross-sectional image parallel to the rotation axis, (*d*) line profiles of the region between arrows *A* and *B* in (*b*) (black line) and arrows *C* and *D* in (*c*) (gray line), respectively. Voxel size is 175 nm × 175 nm (H) × 172 nm (V). Horizontal scan pitch is 175 nm, exposure time is 15 ms and dwell time is 20 ms. Number of projections is 501 for 180° rotation. Total measurement time for tomographic scan is approximately 90 min.

A fossil of a diatom was observed as a test sample to evaluate the spatial resolution of the 3D CT imaging. Since diatoms have fine 3D structures with a size of the order of nanometers, they are very suitable for evaluating the spatial resolution of 3D micro-imaging systems. Fig. 8 ▶ shows phase-contrast CT images of a diatom fossil pasted with glue on a silica glass fiber of diameter 5 µm. Fig. 8(*a*) ▶ shows a 3D rendered image, and Figs. 8(*b*) and 8(*c*) ▶ show virtual cross-sectional images in the horizontal direction (perpendicular to the rotation axis) and in the vertical direction (parallel to the rotation axis), respectively. Fine structures of the fossil are clearly observed. Fig. 8(*d*) ▶ shows line profiles along line regions between arrows *AB* in Fig. 8(*b*) ▶ (black line) and *CD* in Fig. 8(*c*) ▶ (gray line). The line profile *AB* shows that fine structures with a pitch of approximately 0.6 µm are resolved. Therefore, the SIXM-CT system has a spatial resolution of better than 0.6 µm in the horizontal direction. On the other hand, in the vertical direction, indicated by the line profile *CD*, although the signal-to-noise ratio is poorer than in the horizontal direction owing to the streaking noise described in the previous section, it shows almost the same resolution of approximately 0.6 µm.

## 4. Discussion   

In the present status of the SIXM-CT system, one of the problems revealed experimentally is that the vertical phase sensitivity of the CT image is much worse than the horizontal phase sensitivity. This is caused by two types of horizontally streaking background noise. One is white noise seen only in the phase-contrast images [see Figs. 5(*c*) ▶ and 6(*c*) ▶]. The other is ringing noise seen in all the images of absorption contrast, phase contrast and differential phase contrast, especially in the lower region [see Figs. 5(*a*), 5(*b*) ▶, 6(*c*) ▶ and 7(*b*) ▶]. The former is typical in the differential phase-contrast optics. It is due to the phase reconstruction process by linear integration. The integration is applied horizontally line by line to the digital image. Each line integrated has an uncertainty from small errors in pixel values. Although some more robust phase-reconstruction methods with which such streak noise is not generated have already been reported (Arnison *et al.*, 2004 ▶; de Jonge *et al.*, 2008 ▶), these methods are not applicable to the SIXM system because differential phase-contrast data in two orthogonal directions are required. The latter is due to the imaging properties of the imaging optics. In the introduction of the equation of the image signal [equation (3)], the vertical image at the detector plane is assumed to be similar to the transmitted intensity distribution of the object [equation (2′)]. This assumption is, however, strictly valid only in either the full-incoherent illumination system or in the paraxial region of the imaging optics. Otherwise, the image properties are obviously deteriorated by modulated artificial patterns seen as ringing noise. The set-up of the present experiment unfortunately satisfied neither of these requirements; an off-axis condition of the objective and coherent illumination with quasi-parallel beam were employed. For the purpose of the diffraction order selection of the objective FZP, the paraxial region must be blind and full-incoherent illumination is not suitable. This is why the optical system was set with an off-axis condition and coherent quasi-parallel illumination. In Fig. 5 ▶, the lower regions of these images correspond to the larger off-axis distance region of the system. Therefore, ringing noise is more remarkable in the lower region.

In order to solve this problem, employing a partial coherent illumination will be one of the compromises. The largest ringing noise pitch observed in Fig. 5 ▶ was approximately 2 µm. In the case of the present optical system with the focal distance *f* = 100 mm, therefore, the angular divergence of the illuminating beam of roughly larger than 20 µrad will eliminate the ringing noise pattern. On the other hand, however, highly coherent beam is required for the focusing optics to achieve a diffraction-limited spatial resolution. Therefore, an asymmetric coherence condition should be suitable for the SIXM system (Suzuki & Takeuchi, 2007 ▶). Since beam-diffuser (decoherator) and condenser illuminating systems have been proven in the hard X-ray imaging microscope optics (Takeuchi *et al.*, 2009 ▶; Suzuki *et al.*, 2005*b* ▶), these elements dedicated for 1D use will be an effective solution.

## 5. Conclusion   

High-resolution quantitative 3D phase-contrast X-ray imaging has been performed using a SIXM-CT system. The typical measurement time for obtaining a 3D CT image is several hours. The measured spatial resolution of the CT image was better than 0.6 µm. The horizontal phase sensitivity was approximately 80 mg cm^−3^. Imaging properties such as sensitivity, quantitativity and spatial resolution are different between the horizontal plane (perpendicular to the rotation axis) and the vertical plane (parallel to the rotation axis). Vertical image properties are deteriorated by horizontal ringing noise because of the off-axis condition and coherent quasi-parallel illumination of the imaging optics.

Since the SIXM-CT system enables two types of quantitative 3D imaging to be obtained simultaneously, such as phase contrast and absorption contrast, it will open up possibilities of the application in various fields as well as for low-*Z* material samples.
